# Antimicrobial Resistance Phenotypes and Genotypes of *Escherichia coli* Isolates from Artisanal Minas Frescal Cheeses from the Federal District, Brazil

**DOI:** 10.3390/antibiotics14111101

**Published:** 2025-11-02

**Authors:** Letícia Fernandes Silva Rodrigues, Rodrigo Araújo de Melo, Nathalia Mateus Borges, Anna Cléa Silva Aragão, Marta Oliveira de Araújo, Rebeca Dias dos Santos, Carla Azevedo Bilac, Karolina Oliveira Gomes, Bruno Alcântara do Prado, Lívia Cristina Lira de Sá Barreto, Izabel Cristina Rodrigues da Silva, Daniela Castilho Orsi

**Affiliations:** 1 Laboratory of Quality Control and Post-Graduate Program in Health Sciences and Technologies, University of Brasília, Brasília 72220-275, DF, Brazil; rodriguesleticiafs@gmail.com (L.F.S.R.); roodrigodemelo99@gmail.com (R.A.d.M.); nathaliamateus9@gmail.com (N.M.B.); annasilvaaragao@gmail.com (A.C.S.A.); oa.martaaraujo@gmail.com (M.O.d.A.); rebecadias123@gmail.com (R.D.d.S.); gomes.karolina@aluno.unb.br (K.O.G.); prado.bruno@aluno.unb.br (B.A.d.P.); belbiomedica@gmail.com (I.C.R.d.S.); 2 Laboratory of Technologies and Post-Graduate Program in Health Sciences, University of Brasília, Brasília 72220-275, DF, Brazil; carlabilac@gmail.com (C.A.B.); liviabarreto@unb.br (L.C.L.d.S.B.)

**Keywords:** fresh cheese, dairy products, antibiotic resistance, antibiotic resistance genes, *Escherichia coli*

## Abstract

**Background/Objectives:** This study characterized the phenotypic and genotypic profiles of antimicrobial resistance in 104 *Escherichia coli* isolates obtained from 22 samples of artisanal Minas Frescal cheese from the Federal District, Brazil. **Methods**: The antimicrobial susceptibility of *E. coli* isolates was assessed using the disk diffusion method and antimicrobial resistance genes were detected using polymerase chain reaction methods with specific primers. **Results:** The highest rates of phenotypic antimicrobial resistance were observed for sulfonamides (85.58%, 89/104) and tetracyclines (38.46%, 40/104). In the genotypic profiles, most *E. coli* isolates carried the sulfonamide resistance genes *sul1*/*sul2* (62.50%, 65/104), tetracycline resistance genes *tetA*/*tetB* (65.38%, 68/104), and β-lactam resistance genes *blaCTX-M*/*blaTEM*/*blaSHV* (55.77%, 58/104). Most *E. coli* strains that presented sulfonamide resistance genes carried the *sul1* gene (49.04%, 51/104) and were phenotypically sulfonamide-resistant strains (59.61%, 62/104). Regarding the *E. coli* strains that carried tetracycline resistance genes, the majority harbored both *tetA* and *tetB* genes (34.61%, 36/104), with 35.56% (37/104) being phenotypically resistant and 29.80% (31/104) being phenotypically susceptible. For *E. coli* strains that presented β-lactam resistance genes, the most frequently detected gene was *blaCTX-M* (21.15%, 22/104) and, notably, most *E. coli* strains (43.26%, 45/104) were phenotypically susceptible. The *cat1* and *clmA* genes (associated with phenicol resistance) were detected in 22.12% of the *E. coli* isolates (23/104), with only two strains (1.92%) being phenotypically resistant to chloramphenicol. **Conclusion:** The high prevalence of *E. coli* carrying antimicrobial resistance genes in artisanal cheese raises public health concerns regarding the dissemination of potentially pathogenic antimicrobial-resistant microorganisms through the food chain.

## 1. Introduction

Brazil is the largest milk producer in South America, the fourth largest in the world, and one of the top five globally, with an annual production exceeding 34 billion liters [1,2]. Minas Frescal cheese is a typical Brazilian product and ranks third in national cheese consumption, with high availability and low cost in the domestic market [3].

Minas Frescal cheese is characterized by a raw mass with a soft texture, white appearance, and a slightly acidic and salty flavor [4]. It is a fresh cheese, meaning it is not aged, with a high moisture content, medium fat content, and a pH close to neutrality. It is produced through enzymatic coagulation of cow’s milk using rennet and/or other appropriate coagulating enzymes [5,6].

According to Brazilian regulations, the production of Minas Frescal cheese must use milk subjected to pasteurization or an equivalent thermal treatment to ensure the safety of the product. The commercialization of Minas Frescal cheese made with raw milk is not permitted, as the cheese does not undergo a maturation process. Due to its lack of maturation and high moisture content (above 55%), the cheese must be stored at the correct refrigeration temperature (not exceeding 8 °C) and has a short shelf life (approximately 15 days) [5].

In Brazil, Minas Frescal cheese is industrially produced in dairy factories using pasteurized milk and is sold in supermarkets in refrigerated shelves and in packaging containing the seal of the sanitary inspection service. However, the Brazilian market also includes artisanal Minas Frescal cheeses, which are typically produced on small rural properties and commonly sold at open-air markets and farmers’ markets. These cheeses are often sold without branding on the packaging, without refrigeration, and are frequently made from raw milk and handled under inadequate hygienic conditions, making them susceptible to a high presence of pathogenic microorganisms, which compromises their quality and poses a food safety risk to consumers. The main pathogens isolated from these cheeses are *Escherichia coli*, *Staphylococcus aureus*, *Salmonella* spp., and *Listeria monocytogenes*, which frequently cause outbreaks of foodborne diseases worldwide [7,8,9,10,11].

Some studies have shown that artisanal cheeses produced in different regions of Brazil do not meet the microbiological criteria established by national laws, mainly due to high levels of *S. aureus* and *E. coli* [8,11,12]. *Escherichia coli* predominantly lives as a commensal bacterium in the intestinal microbiota of humans and homeothermic animals. However, pathogenic strains of *E. coli* can cause various diseases, with these bacteria being one of the main causes of gastrointestinal infections in humans, primarily transmitted through contaminated water and food. Consequently, these bacteria are abundant in feces and are regularly used as microbial indicators of the presence of enteropathogenic bacteria in water and food [7,10,13].

*E. coli* exhibits a high prevalence of antimicrobial resistance, both in commensal strains and in those that possess virulence factors. Therefore, it is essential to monitor the antimicrobial resistance profile of *E. coli* due to its widespread presence in the environment and among various hosts, as well as its ability to transfer resistance genes both inter- and intra-specifically. Consequently, *E. coli* is commonly used as a microorganism to monitor antimicrobial resistance, given its capacity to acquire resistance genes through horizontal transfer [14,15].

Thus, this study aimed to isolate *Escherichia coli* strains from artisanal Minas Frescal cheese samples collected at farmers’ markets in the Federal District, Brazil. The *E. coli* strains were genetically confirmed through the detection of the *uidA* gene. Antimicrobial susceptibility was assessed using the disk diffusion method (Kirby–Bauer). Recent studies have emphasized the importance of determining the genotypic profiles of antimicrobial resistance in both phenotypically resistant and susceptible bacteria, as many carry silent genes that are not expressed in the phenotypic profile but can be spread through horizontal gene transfer to other bacteria and become active in the new host [16,17,18,19,20]. Therefore, while many studies only determine the genotypic profile for *E. coli* strains that are phenotypically resistant to antimicrobials isolated from milk and cheeses [21,22,23], the present study investigated the presence of resistance genes in all *E. coli* strains (phenotypically resistant or susceptible to antimicrobials), including *sul1* and *sul2* (resistance to sulfonamides); *tetA* and *tetB* (resistance to tetracyclines); *blaCTX-M*, *blaTEM*, and *blaSHV* (resistance to β-lactams); and *cat1* and *clmA* (resistance to phenicols).

## 2. Results and Discussion

### 2.1. Isolation of E. coli from Cheese Samples and Determination of the Phenotypic Profile of Antimicrobial Resistance

The results of this study showed that all samples of artisanal Minas Frescal cheese contained thermotolerant coliforms and *E. coli*, and 31.8% of artisanal cheeses (7/22) exceeded the limit imposed by Brazilian legislation of 3.0 log MPN/g for thermotolerant coliforms [24]. Thermotolerant coliforms are a subgroup of total coliforms that ferment lactose at temperatures between 44.5 and 45.5 °C, with the main representative being the *E. coli* species, which is exclusively of fecal origin [8]. Food contaminated with *E. coli* is not strictly associated with foodborne diseases, as this bacterium can commensally inhabit the intestines of humans and various other animals. However, the presence of pathogenic *E. coli* serotypes in food can cause gastrointestinal illnesses in consumers [13,25].

A total of 104 *E. coli* strains were isolated from the 22 samples of artisanal Minas Frescal cheese, with genetic confirmation of the *uidA* and *lacZB* genes. The *uidA* gene is used for *E. coli* confirmation, as it is present in more than 95% of the strains of this bacterium but absent in other *Enterobacteriaceae* species, making it a specific gene for the detection of *E. coli* [26,27].

Ahmady et al. [28] isolated 76 suspected *E. coli* strains from raw cow milk and unpasteurized butter samples collected from dairy stores in Ahvaz, southwest Iran, detecting the *uidA* gene in 65.9% (50/76) of the strains, which were confirmed to be *E. coli*. Similarly, Alsanjary et al. [29] analyzed 400 bacteria collected from various dairy herds in Nineveh, Iraq, and observed that 35.0% (140/400) of the strains tested positive for *E. coli* through the identification of the *uidA* gene.

Table 1 presents the antimicrobial susceptibility profile of the 104 *E. coli* strains isolated from artisanal Minas Frescal cheese. The highest antimicrobial resistance rates were observed for sulfonamide (85.58%, 89/104) and tetracycline (38.46%, 40/104).

In the literature, several studies have reported high antimicrobial resistance rates to sulfonamides and tetracyclines in *E. coli* isolated from raw milk and cheeses. Ribeiro et al. [30] reported that 93.0% of potentially pathogenic *E. coli* strains isolated from raw milk samples in a small Brazilian farm producing fresh raw milk cheese were resistant to sulfamethoxazole/trimethoprim. Messele et al. [31] analyzed 224 raw milk samples collected from cows with mastitis at dairy farms in central Ethiopia and found an *E. coli* prevalence rate of 7.1% (16), with a phenotypic resistance rate of 50% to sulfamethoxazole/trimethoprim. Shoaib et al. [22] analyzed 209 samples collected from a large dairy farm in Xinjiang province, China, and found that most of the 338 *E. coli* strains were resistant to sulfamethoxazole/trimethoprim (62.43%, 211/338) and exhibited a tetracycline resistance rate of 28.99% (98/338). Hassanien and Shaker [32] found *E. coli* O157:H7 strains in 11.3% of dairy product samples, such as cheeses and yogurts, sold in Egypt, and these strains exhibited high resistance to tetracycline (81.8%). Joubrane et al. [33] analyzed 195 raw milk samples collected in Lebanon; among the 100 isolated *E. coli* strains, 41.0% showed resistance to tetracycline. De Campos et al. [7] reported that, out of a total of 147 samples of Minas Frescal cheese made from unpasteurized cow’s milk in Brazil, 39 *E. coli* strains were isolated, with the highest antimicrobial resistance rate found for tetracycline (25.6%).

Antimicrobials such as tetracyclines and sulfonamides are widely used in the treatment of animal diseases. According to the USA’s Food and Drug Administration, in 2021, the estimated proportion of antimicrobial drugs sold for use in food-producing animals, based on therapeutic class, was 67% for tetracyclines, representing the highest sales volume in the domestic market, with approximately 3916 kg of drugs sold. This was followed by penicillins at 10% (619 kg), macrolides at 9% (524 kg), and sulfonamides at 5% (302 kg) [34].

It was observed that among the 104 *E. coli* strains, only 13 (12.5%) were sensitive to all tested antimicrobials, while 91 (87.5%) exhibited resistance to at least one of the tested antimicrobials. A total of 33 *E. coli* strains (31.8%) were identified as resistant to three or more classes of antimicrobials and were therefore classified as multidrug-resistant (MDR) bacteria. A total of 31 antimicrobial resistance profiles were identified (Table 2), with resistance to SUL being the most frequent, being observed in 32.7% of *E. coli* strains (34/104), followed by resistance to SUL-TET in 7.7% (8/104) and SUL-TET-CIP in 5.8% (6/104).

Several studies from different regions have reported a considerable prevalence of multidrug-resistant (MDR) *E. coli* in dairy products. In Egypt, Kasem et al. [35] observed that 82.4% of 17 *E. coli* isolates from commonly consumed cheese varieties were MDR. In Lebanon, Hussein et al. [36] found that 75.0% (89/118) of *E. coli* strains from 50 white soft cheese (Akkawi) samples collected from 16 major retail stores in Beirut exhibited multidrug resistance, particularly in unbranded products lacking proper pasteurization. In Ethiopia, Messele et al. [31] reported MDR in 68.7% of *E. coli* isolates obtained from raw milk sampled at central-region dairy farms, while Adzitey et al. [37] identified MDR in 40.5% (42/250) of *E. coli* strains recovered from raw cow milk and related products collected from different locations in the Saboba district of Ghana.

Multidrug-resistant *E. coli* has become a significant concern in human and veterinary medicine. Dairy cattle may serve as reservoirs for zoonotic and antibiotic-resistant strains, facilitating their spread through contaminated farm environments, milk, meat, or direct contact with animals [14,22].

### 2.2. Determination of the Genotypic Profile of Antimicrobial Resistance of E. coli Isolates

In the present study, 65 *E. coli* strains (62.50%) out of the 104 analyzed carried sulfonamide resistance genes (Table 3). Most of the isolated *E. coli* strains harbored the *sul1* gene (57.69%, 60/104), with a large proportion of them carrying this gene alone (49.04%, 51/104). In total, 14 *E. coli* strains (13.46%) carried the *sul2* gene, of which only 5 *E. coli* strains (4.81%) carried *sul2* alone. Accordingly, nine *E. coli* strains (8.65%) harbored both *sul1* and *sul2* genes simultaneously. In the antibiogram analysis, most *E. coli* strains were resistant to sulfonamide (85.58%, 89/104), and in the genotypic profile, 62 (59.61%) of these phenotypically sulfonamide-resistant *E. coli* strains carried the *sul1* and/or *sul2* genes. This result indicates that 69.66% of phenotypically sulfonamide-resistant *E. coli* strains carried the *sul1* and/or *sul2* genes. Furthermore, a small proportion of *E. coli* strains (2.88%, 3/104) were phenotypically susceptible to sulfonamide but still harbored the *sul1* and/or *sul2* genes.

Sulfonamides exhibit a broad spectrum of activity against most Gram-positive and Gram-negative bacteria, acting through the competitive inhibition of the enzyme dihydropteroate synthase (DHPS), which is essential for folic acid synthesis in bacteria. Folic acid is crucial for the construction of bacterial DNA and RNA; therefore, inhibiting its synthesis prevents bacterial growth and replication. Sulfonamides have been used for decades in animals and humans and sulfonamide resistance mechanisms have been frequently identified in Gram-negative bacteria, mainly due to the acquisition of three resistance genes (*sul1*, *sul2*, and *sul3*), which encode DHPS isoforms with low affinity for sulfonamides. These resistance genes have been identified in both bacterial chromosomes and plasmids, often associated with mobile genetic elements such as transposons and integrons. This genetic mobility facilitates the transfer of *sul* genes, contributing to the spread of resistance among different bacterial populations [38,39,40].

The *sul1* and *sul2* genes are frequently detected in *E. coli*, while the *sul3* gene is much less common. The *sul1* gene is highly prevalent due to its location within the 3-conserved segment of class 1 integrons. Consequently, it is frequently co-located with other antimicrobial resistance genes carried on gene cassettes in the variable region of these integrons. Class 1 integrons containing *sul1* have been detected in *E. coli* from both healthy and diseased food-producing animals worldwide. The *sul2* gene is also widely distributed among *E. coli* isolates from different animal species across various regions of the world [38,39].

Ombarak et al. [21] evaluated 25 *E. coli* isolates phenotypically resistant to sulfonamides from samples of raw milk and the two most popular cheeses in Egypt and found that 25 strains (100%) carried the *sul2* gene, 7 strains (28%) carried *sul1*, and 3 strains (12%) carried *sul3*. Shoaib et al. [22] isolated 211 *E. coli* strains resistant to trimethoprim/sulfamethoxazole from a dairy farm environment in Xinjiang, China, and observed that 142 strains (67.3%) carried *sul2*, 59 strains (27.9%) carried *sul1*, and 38 strains (18.1%) carried *sul3*. Kuzeubayeva et al. [41] analyzed 207 samples of three types of cheese produced in Kazakhstan and observed that 31.4% of the cheese samples were contaminated with *E. coli*. The samples of soft cheese produced by small farms (80% of the samples) and packaged at the retail site (100%) showed the highest level of contamination. A total of 65 *E. coli* strains were isolated, of which 20 strains (30.8%) carried the *sul1* gene.

It was observed that 68 *E. coli* strains (65.38%) out of the 104 isolates carried tetracycline resistance genes (Table 3). Among these, 58 *E. coli* strains (85.29%) harbored the *tetA* gene, with 22 *E. coli* strains (21.15%) carrying only this gene. Additionally, 46 *E. coli* strains (67.64%) carried the *tetB* gene, with only 10 *E. coli* strains (9.61%) carrying this gene exclusively. Finally, 36 *E. coli* strains (34.61%) simultaneously harbored both *tetA* and *tetB* genes. Regarding the phenotypic profile, 40 *E. coli* strains (38.46%) exhibited tetracycline resistance, while in the genotypic profile, 37 of the resistant *E. coli* strains (35.56%) carried the *tetA* and/or *tetB* genes. Therefore, 92.5% of *E. coli* strains resistant to tetracycline in the antibiogram carried the *tetA* and/or *tetB* genes. Additionally, among the 104 *E. coli* strains analyzed, 31 (29.80%) were susceptible to tetracycline in the antibiogram, but still carried the *tetA* and/or *tetB* genes.

Tetracycline resistance is widespread in *E. coli* from livestock, mainly mediated by efflux pumps encoded by *tet* genes. Among these, *tetA* and *tetB* are the most common, often carried on plasmids associated with mobile genetic elements such as transposons and integrons [42,43,44,45].

Several studies in the literature have reported the presence of *tetA* and *tetB* genes in *E. coli* isolated from raw milk and cheeses. Ombarak et al. [21] evaluated 61 *E. coli* isolates from 187 samples of raw milk and the two most popular cheeses in Egypt, all phenotypically resistant to tetracycline, and found that 53 strains (86.9%) carried the *tetA* gene, 9 (14.8%) carried *tetB*, 1 (1.63%) carried *tetD*, and none carried *tetC*. Shoaib et al. [22] analyzed 98 *E. coli* strains isolated from a large dairy farm in Xinjiang province, China, all phenotypically resistant to tetracycline, and found a higher presence of the *tetB* gene in 69 *E. coli* strains (70.4%), followed by *tetA* in 11 *E. coli* strains (11.2%), and no presence of the *tetD* gene. Belaynehe et al. [23] reported that 88 *E. coli* isolates (95.7%) from cattle farms, out of a total of 92 tetracycline-resistant isolates, carried tetracycline resistance genes. Among them, 47 *E. coli* isolates (51.1%) harbored the *tetA* gene, while 41 (44.6%) harbored *tetB*. Messele et al. [31] analyzed 16 *E. coli* strains isolated from raw milk samples from cows with mastitis at dairy farms in central Ethiopia and found that eight isolates (50.0%) carried the *tetA* gene. Tabaran et al. [46] isolated 27 enterotoxigenic *E. coli* (ETEC) and verotoxigenic *E. coli* (VTEC) strains from 120 raw milk samples and 80 unpasteurized cheese samples sold in Romania. Among these, 48.1% of *E. coli* strains (13/27) tested positive for tetracycline resistance genes. The *tetA* and *tetB* genes were identified in 38.5% of *E. coli* strains (5/13), *tetC* was identified in 30.8% (4/13), and 23.1% of *E. coli* strains (3/13) were positive for *tetA*, *tetB*, and *tetC*.

A notable finding in this study was the presence of β-lactam antimicrobial resistance genes (*blaCTX-M*, *blaTEM*, and *blaSHV*) in 58 *E. coli* strains (55.77%) (Table 3). However, lower resistance rates were observed in the phenotypic profile for the tested β-lactam antimicrobials: 19.23% (20/104) for amoxicillin/clavulanic acid, 13.46% (14/104) for cefotaxime, and 10.58% (11/104) for ceftazidime. Thus, most *E. coli* strains (43.26%, 45/104) that carried β-lactam resistance genes were susceptible in the antibiogram. Meanwhile, 20 *E. coli* strains (19.23%) were resistant in the antibiogram to amoxicillin/clavulanic acid and/or cefotaxime and/or ceftazidime and carried β-lactam antimicrobial resistance genes.

The most frequently detected β-lactam antimicrobial resistance gene in *E. coli* strains was *blaCTX-M* (21.15%, 22/104). Additionally, 14 *E. coli* strains (13.46%) simultaneously carried the *blaCTX-M* and *blaTEM* genes, while 10 (9.61%) harbored only the *blaSHV* gene. The *blaTEM* gene was found in nine strains (8.65%), whereas five (4.81%) carried all three genes (*blaCTX-M*, *blaTEM*, and *blaSHV*). Finally, three strains (2.88%) presented a combination of *blaCTX-M* and *blaSHV* genes, and another three (2.88%) carried a combination of *blaSHV* and *blaTEM* genes.

Gram-negative bacteria, especially species such as *Escherichia coli* and *Klebsiella pneumoniae* from the Enterobacteriaceae family, which produce extended-spectrum β-lactamases (ESBLs), are among the main causes of resistance to β-lactam antimicrobials. ESBL-producing bacteria are resistant to penicillin and its derivatives, as well as first-, second-, and third-generation cephalosporins and monobactams. This resistance is mainly due to the production of CTX-M, TEM, and SHV β-lactamases, which are encoded by the *bla-CTX-M*, *bla-SHV*, and *bla-TEM* genes, respectively. The genes encoding ESBLs are frequently located on plasmids and housed within transposons, facilitating their spread among human and animal hosts. This phenomenon is a significant concern as it compromises the effectiveness of commonly used antimicrobials in the treatment of bacterial infections [47,48,49].

Several studies in the literature have reported a high prevalence of β-lactam antibiotic resistance genes in *E. coli* strains isolated from milk and dairy products. Shoaib et al. [22] isolated 263 *E. coli* strains resistant to cefotaxime from a dairy farm environment in Xinjiang, China, and observed that 148 strains (56.3%) carried the *blaTEM* gene, 68 (25.8%) carried *blaOXA*, and 59 (22.4%) carried *blaCTX-M*. Eldesoukey et al. [50] examined a total of 240 samples (75 from diarrheic calves, 150 from milk samples, and 15 from workers) for the prevalence of EPEC in three dairy farms in Egypt, and 28 EPEC isolates were detected. Among these 28 EPEC isolates, 5 strains (17.90%) carried the *blaSHV* gene, 3 (10.70%) carried both the *blaTEM* and *blaCTX-M* genes, 2 strains (7.10%) carried *blaTEM*, and 1 strain (3.60%) carried *blaCTX-M*, totaling 11 EPEC strains (39.30%) with the presence of the studied genes.

Ombarak et al. [21] reported that among 42 *E. coli* isolates resistant to ampicillin, obtained from 187 samples of raw milk and the two most popular cheeses in Egypt, 40 isolates (94.23%) carried the *blaTEM* gene, 9 (21.42%) carried *blaCTX-M*, and 3 (7.14%) carried *blaSHV*. In the same study, 10 *E. coli* isolates resistant to ampicillin were identified as ESBL producers, of which 9 (90.00%) carried the *blaCTX-M* gene, 8 (80.00%) carried *blaTEM*, and 1 (10.00%) carried *blaSHV*. Among these, five ESBL-producing isolates (50.00%) carried both the *blaCTX-M* and *blaTEM* genes. Tabaran et al. [46] reported that, out of 27 pathogenic *E. coli* strains isolated from 120 raw milk samples and 80 unpasteurized cheese samples sold in Romania, 33.3% (n = 9) carried the beta-lactamase gene *blaTEM* and none of the samples tested positive for *blaSHV*.

In this study, the *cat1* and *clmA* genes (associated with resistance to phenicols) were detected in 23 *E. coli* isolates (22.12%), with only 2 strains (1.92%) being phenotypically resistant to chloramphenicol. Most of the strains (20.19%, 21/104) were phenotypically sensitive to chloramphenicol yet still carried resistance genes to phenicols (Table 3). It was observed that most of the *E. coli* strains carried the *clmA* gene (10/104, 17.31%), while 4 *E. coli* (3.85%) carried *cat1*, and 1 (0.96%) carried both *cat1* and *clmA*.

Phenicols are broad-spectrum antimicrobials commonly used in veterinary practice. Due to the severe toxicity of chloramphenicol, which can lead to life-threatening blood disorders such as irreversible aplastic anemia, hypoplastic anemia, thrombocytopenia, and granulocytopenia, its use in food-producing animals was prohibited in the European Union in 1994 and in Brazil in 2003. However, the fluorinated derivative florfenicol remains approved for treating bacterial infections in livestock [14,51].

A total of 60 genotypic antimicrobial resistance patterns of *E. coli* isolates were identified (Table 4), representing 80.77% of the isolated *E. coli* strains (84/104). The most frequent genotypic antimicrobial resistance patterns were *blaCTX-M–sul1–tetA–tetB–blaTEM*, observed in 4.81% of the strains (5/104), and *sul1–tetA–tetB*, observed in 3.85% of the strains (4/104).

In the present study, seven *E. coli* strains were found to exhibit a resistant phenotype without carrying any of the resistance genes investigated. These strains likely harbor other antimicrobial resistance genes not included in the current analysis. On the other hand, several *E. coli* isolates did not show resistance in the antibiogram but expressed resistance genes in PCR. Recent studies have been published referring to these genes as ‘silent genes’ [16,17,18,19,20].

Silent genes are DNA sequences that are usually inactive or expressed at very low levels but can be activated by mutations, recombination, or transfer to a new host [16]. Some genes may appear silent in the laboratory yet are expressed in natural environments. Like other genes, silent genes can spread via horizontal gene transfer. Studying the full resistome, including silent genes, is crucial for understanding antibiotic resistance, as both resistant and phenotypically susceptible strains should be considered [16,17,18,19,20].

**Figure 1:**
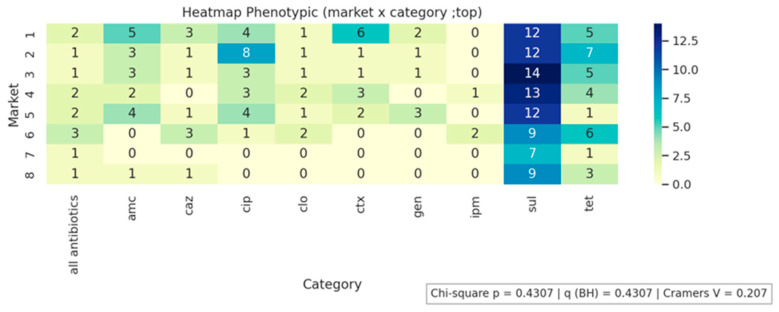
Phenotypic resistance distribution across markets.

**Figure 2:**
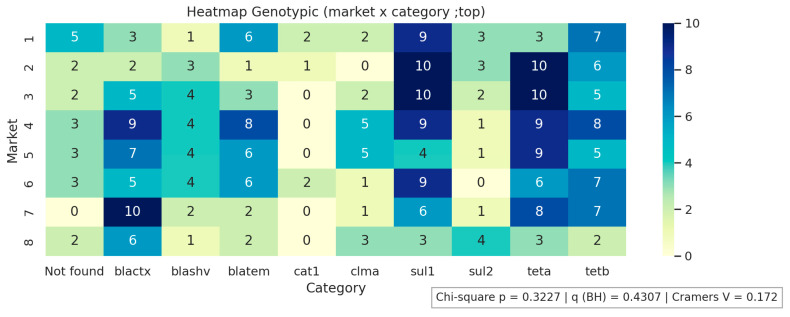
Genotypic resistance distribution across markets. Not found indicates that none of the investigated genes were detected.

**Figure 3:**
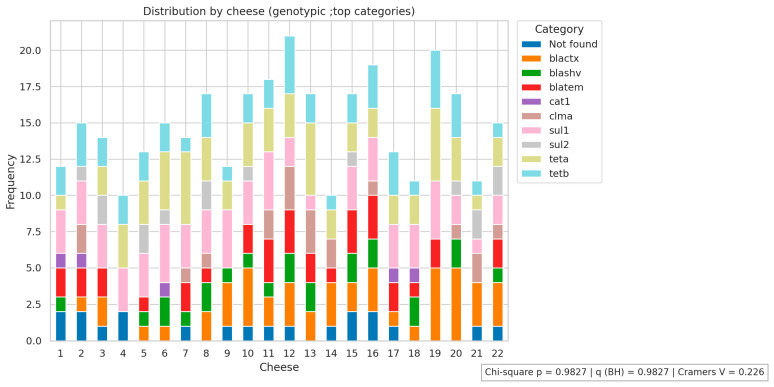
Antimicrobial resistance genes distribution across cheese samples. Not found indicates that none of the investigated genes were detected.

Figure 1, Figure 2 and Figure 3 present the analyses of phenotypic and genotypic resistance categories across markets and, consequently, cheese samples. The associations between markets and phenotypic (Figure 1) and genotypic (Figure 2) resistance categories were weak, with small effect sizes and non-significant chi-square values (*p* = 0.43 and *p* = 0.32, respectively), indicating that resistance profiles were broadly distributed among the markets and their corresponding cheese samples, without apparent geographical differences in antimicrobial resistance patterns. The distribution of antimicrobial resistance genes across cheese samples (Figure 3) also showed limited evidence of dependence (*p* = 0.98), suggesting that resistance genes were not clustered in specific markets or cheese samples. Overall, these results indicate that phenotypic and genotypic resistance traits are dispersed across the sampled markets, and there is no significant concentration of resistance patterns in particular locations or products.

All antibiotics indicates susceptibility to all tested antibiotics. AMC, amoxicillin/clavulanic acid; CAZ, ceftazidime; CIP, ciprofloxacin; CLO, chloramphenicol; CTX, Cefotaxime; GEN, gentamicin; IMP, imipenem; SUL, sulfonamide; TET, tetracycline

Minas frescal cheese has intrinsic factors, such as high moisture, neutral pH, and nutrient richness, which favor bacterial growth. This, combined with the use of raw milk in its production, results in a high level of contamination with potentially pathogenic bacteria, including *E. coli*. The situation is further aggravated by the presence of both genotypic and phenotypic antimicrobial resistance in these bacteria. Therefore, it is essential to implement educational programs for small-scale artisanal cheese producers, emphasizing the prohibition of using raw milk in the production of fresh cheeses, and strengthening market surveillance of these products. Such measures are fundamental to improving food safety and protecting consumers from potential health risks associated with the consumption of these cheeses [7,8,10,11,12].

## 3. Material and Methods

### 3.1. Sample Collection

A total of 22 samples of artisanal Minas Frescal cheese were collected from 8 different farmers’ markets in the Federal District, Brazil, between February 2022 and March 2024, with 2 to 3 samples from different vendors collected at each market. The samples were immediately transported to the laboratory in a thermal box containing ice, and microbiological analyses began a maximum of one hour after collection. All samples were analyzed in triplicate, meaning three aliquots were taken from each package, and the results were expressed as means and standard deviations.

### 3.2. Enumeration of Thermotolerant Coliforms and Isolation of E. coli

Enumeration of thermotolerant coliforms and isolation of *E. coli* were performed according to the methods described by Feng et al. [52]. For the analysis, 25 g of each sample was weighed and diluted in 225 mL of 0.1% peptone water (*w*/*v*). The material was homogenized, resulting in the first dilution (10^−1^). Subsequent decimal dilutions (up to 10^−3^) were prepared from this initial dilution. The determination of the Most Probable Number (MPN) of thermotolerant coliforms was conducted using the multiple-tube fermentation technique, starting with the presumptive test. This involved inoculating each sample dilution into Lauryl Sulfate Tryptose Broth (HiMedia, Thane, India). The tubes were incubated at 37 °C for 24 h. A positive result was indicated by turbidity in the broth accompanied by gas production in the Durham tubes. Aliquots from the positive tubes in the presumptive test were inoculated into tubes containing *Escherichia coli* broth (EC broth, Kasvi, Madrid, Spain) to confirm the presence of thermotolerant coliforms. The tubes were incubated in a water bath at 45 °C for 24 h. Again, a positive result was indicated by turbidity accompanied by gas production. The results were expressed as log MPN/g. For the isolation of *E. coli*, aliquots of the EC broth were streaked onto MacConkey Agar (HiMedia, Thane, India), and the plates were incubated at 37 °C for 24 h. Subsequently, 6 strains suspected of being *E. coli* (strongly lactose-fermenting colonies) were isolated from each sample, each presenting distinct antibiograms, totaling 132 suspected strains. These strains were subjected to molecular identification using the polymerase chain reaction (PCR) technique to confirm the presence of *E. coli*. After genetic identification, 104 strains were confirmed as *E. coli*. The *uidA* gene was not amplified in 28 strains; therefore, these strains were discontinued.

### 3.3. Bacterial DNA Extraction

The isolated bacterial colonies were cultured in Mueller–Hinton broth (Kasvi Madrid, Spain) for 18–24 h. Then, DNA extraction was performed using the Purelink Genomic DNA Mini Kit Invitrogen™ (Thermo Fisher Scientific, Waltham, MA, USA), following the manufacturer’s protocol for Gram-negative bacteria. The quality of the extracted DNA was assessed via electrophoresis on a 2% (*w*/*v*) agarose gel, and the DNA concentration was quantified using the NanoDrop 2000 (Thermo Fisher Scientific, Pittsburgh, PA, USA). The DNA products were diluted with Milli-Q water to an average concentration of 20 ng/μL.

### 3.4. Identification of E. coli

For the identification of *E. coli*, the *uidA* gene, which encodes the enzyme β-glucuronidase, and the *lacZB* gene, which encodes the enzyme β-galactosidase, were used as characteristic markers of the species [27]. The primers sequences are detailed in Table 5.

For the *uidA* gene, the PCR thermocycling conditions were as follows: initial denaturation at 95 °C for 2 min, 35 cycles of denaturation at 95 °C for 1 min, annealing at 60 °C for 1 min, and extension at 72 °C for 1 min. For the *lacZB* gene, the PCR thermocycling conditions were initial denaturation at 94 °C for 5 min, 35 cycles of denaturation at 94 °C for 1 min, annealing at 60 °C for 1 min, and extension at 72 °C for 1 min, followed by a final extension at 72 °C for 8 min. Gene fragment amplification was carried out using the following thermocyclers: Life Express Thermal Cycler, model TC-96/G/H(b); Swift MiniPro Thermal Cycler, model SWT-MIP-0-2-1; and Life Touch Thermal Cycler, model TC-96/G/H(b)B. The PCR products were subjected to electrophoresis on 2% (*w*/*v*) agarose gel (Invitrogen Life Technologies, Carlsbad, CA, USA), stained with ethidium bromide (Sigma-Aldrich-Merck, Darmstadt, Germany), at a power setting of 50 W. The results were visualized under ultraviolet (UV) light using a 100 bp DNA ladder molecular weight marker (Ludwig Biotecnologia, Porto Alegre, Brazil).

### 3.5. Antimicrobial Susceptibility Profile of E. coli

The antimicrobial susceptibility test for *E. coli* strains was performed using the standard Kirby–Bauer disk diffusion method [53]. The bacterial inoculum was prepared by a direct suspension of microbial growth in Mueller–Hinton broth, adjusted to a turbidity equivalent to the 0.5 McFarland standard. The microbial inoculum was spread uniformly on the surface of a Mueller–Hinton agar plate using a sterile swab. After the inoculum dried, antimicrobial agent disks were applied. Results were obtained after 24 h of incubation at 37 °C by measuring the inhibition zone diameters in millimeters. The antimicrobials and reference values for interpreting inhibition zones are detailed in Table 6. *E. coli* showing resistance to three or more antimicrobial agents from different classes were classified as multidrug-resistant (MDR).

Antimicrobial drugs were selected based on different classes and their importance for veterinary use (mainly in dairy cattle farming) and/or their relevance for human medicine. Thus, the drugs with the highest veterinary use include tetracyclines followed by the penicillin class (such as amoxicillin combined with clavulanic acid), the phenicol class (although chloramphenicol is banned, the fluorinated derivative florfenicol is licensed for the treatment of bacterial infections in food-producing animals), and the sulfonamide class (sulfonamides). On the other hand, the use of drugs such as ciprofloxacin (quinolones) and cefotaxime and ceftazidime (cephalosporins) in animal disease treatment is more restricted and controlled due to their critical importance in human medicine [54,55]. Although gentamicin (aminoglycoside) is permitted for veterinary use in Brazil, as in human medicine, its use is limited due to the risk of toxicity [56,57]. Finally, imipenem (carbapenem) is prohibited for use in animal treatment due to its critical importance in human medicine, where it is considered a last-resort antibiotic [58].

### 3.6. Detection of Antimicrobial Resistance Genes in E. coli

All 104 *E. coli* strains were examined for the presence of the following antimicrobial resistance genes: *sul1* and *sul2* (resistance to sulfonamides); *tetA* and *tetB* (resistance to tetracyclines); *blaCTX-M*, *blaTEM*, and *blaSHV* (resistance to β-lactams); and *cat1* and *clmA* (resistance to chloramphenicol). The primer sequences and PCR conditions are described in Table 7. Gene amplification was carried out using the following thermocyclers: Life Express Thermal Cycler, model TC-96/G/H(b); Swift MiniPro Thermal Cycler, model SWT-MIP-0-2-1; and Life Touch Thermal Cycler, model TC-96/G/H(b)B. For agarose gel electrophoresis, 8 microliters of the PCR amplification products, supplemented with 2 µL of bromophenol blue (Dinâmica Química, São Paulo, Brazil), was subjected to separation using 2% (*w*/*v*) agarose gel electrophoresis (Invitrogen Life Technologies, Carlsbad, CA, USA) under a constant voltage of 50 V. The DNA fragments were stained with ethidium bromide (Sigma-Aldrich-Merck, Darmstadt, Germany) and visualized under ultraviolet (UV) light. A 100 bp marker (Ludwig Biotecnologia, Porto Alegre, Brazil) was used as a molecular weight reference standard.

### 3.7. Statistical Analysis

To investigate potential associations between phenotypic and genotypic resistance categories across markets and cheeses, chi-square tests of independence were performed on the main categories. Cramér’s V was computed to estimate effect sizes, and *p*-values were adjusted using the Benjamini–Hochberg procedure. The analyses indicated associations between market and resistance categories, as well as patterns of phenotypic and genotypic resistance distribution across cheeses. Statistical analyses were conducted in Python (version 3.11; Python Software Foundation, Wilmington, DE, USA). 

## 4. Conclusions

In this study, a total of 104 *E. coli* strains were isolated from 22 samples of artisanal Minas Frescal cheese, with the highest rates of phenotypic antimicrobial resistance observed for sulfonamides (85.58%, 89/104) and tetracyclines (38.46%, 40/104). In the genotypic profiles, most *E. coli* isolates carried the sulfonamide resistance genes *sul1* and/or *sul2* (62.50%, 65/104), the tetracycline resistance genes *tetA* and/or *tetB* (65.38%, 68/104), and the β-lactam resistance genes *blaCTX-M*, *blaTEM*, and/or *blaSHV* (55.77%, 58/104). The high prevalence of *E. coli* strains exhibiting both phenotypic and genotypic antimicrobial resistance in artisanal Minas Frescal cheese raises public health concerns due to the potential dissemination of these resistance determinants through the food chain. These findings highlight the need for stricter regulation and more effective oversight of the production and commercialization processes of these artisanal cheeses, as well as the implementation of educational strategies for producers regarding the microbiological risks associated with these products.

## Figures and Tables

**Table 1:** Antimicrobial susceptibility profile of *Escherichia coli* isolates.

Antimicrobials	R % (n)	I % (n)	S % (n)
Amoxicillin * (AMC)	19.23 (20)	3.85 (4)	76.92 (80)
Cefotaxime (CTX)	13.46 (14)	6.73 (7)	79.81 (83)
Ceftazidime (CAZ)	10.58 (11)	5.77 (6)	83.65 (87)
Ciprofloxacin (CIP)	23.08 (24)	18.27 (19)	58.65 (61)
Chloramphenicol (CLO)	7.69 (8)	2.88 (3)	89.43 (93)
Gentamicin (GEN)	8.65 (9)	19.23 (20)	72.12 (75)
Imipenem (IMP)	2.88 (3)	3.85 (4)	93.27 (97)
Sulfonamide (SUL)	85.58 (89)	4.81 (5)	9.61 (10)
Tetracycline (TET)	38.46 (40)	2.88 (3)	58.65 (61)

* Amoxicillin and clavulanic acid; R: resistant; I: intermediate; S: susceptible; n = number of strains; % = percentage of the total of 104 strains.

**Table 2:** Phenotypic antimicrobial resistance patterns of *Escherichia coli* isolates.

Isolates	Antibiotics	n (%)
1	SUL	34 (32.7)
2	TET	2 (1.92)
3	SUL–AMC	1 (0.96)
4	SUL–CAZ	3 (2.88)
5	SUL–CIP	4 (3.86)
6	SUL–CLO	1 (0.96)
7	SUL–CTX	1 (0.96)
8	SUL–GEN	3 (2.88)
9	SUL–TET	8 (7.69)
10	SUL–IMP	1 (0.96)
11	SUL–CTX–CIP	1 (0.96)
12	SUL–TET–CIP	6 (5.76)
13	SUL–AMC–CIP	2 (1.92)
14	SUL–CTX–AMC	1 (0.96)
15	SUL–TET–AMC	2 (1.92)
16	SUL–GEN–CIP	1 (0.96)
17	SUL–CTX–TET–AMC	2 (1.92)
18	SUL–CLO–AMC–CIP	1 (0.96)
19	SUL–CTX–AMC–CAZ	3 (2.88)
20	SUL–CLO–TET–CIP	1 (0.96)
21	SUL–TET–AMC–CIP	2 (1.92)
22	SUL–TET–GEN–CIP	1 (0.96)
23	SUL–CLO–TET–AMC	1 (0.96)
24	SUL–CTX–TET–AMC–CIP	1 (0.96)
25	SUL–CLO–CTX–TET–CIP	1 (0.96)
26	SUL–CLO–TET–IPM–CAZ	2 (1.92)
27	SUL–CTX–TET–GEN–CAZ	1 (0.96)
28	SUL–TET–GEN–AMC–CIP	1 (0.96)
29	SUL–CTX–TET–AMC–CIP–CAZ	1 (0.96)
30	SUL–CTX–TET–GEN–AMC–CIP	1 (0.96)
31	SUL–CLO–CTX–TET–GEN–AMC–CAZ	1 (0.96)
Total		91 (87.5)

n = number of strains; % = percentage of the total of 104 strains. AMC, amoxicillin/clavulanic acid; CTX, Cefotaxime; CAZ, ceftazidime; CIP, ciprofloxacin; CLO, chloramphenicol; GEN, gentamicin; IMP, imipenem; SUL, sulfonamide; TET, tetracycline.

**Table 3:** Percentage occurrence of various antibiotic resistance genes in 104 *Escherichia coli* isolates.

Antimicrobial Resistance Genes	Total of *E. coli* Strains n (%)	Phenotypically Resistant *E. coli* n (%)	Phenotypically Susceptible *E. coli* n (%)
	*Sul* genes		
*sul1*	51 (49.04)	48 (45.15)	3 (2.88)
*sul2*	5 (4.81)	5 (4.81)	0
*sul1* + sul2	9 (8.65)	9 (8.65)	0
Total *sul* genes	65 (62.50)	62 (59.61)	3 (2.88)
	*Tet* genes		
*tetA*	22 (21.15)	11 (10.57)	11 (10.57)
*tetB*	10 (9.61)	3 (2.88)	7 (6.73)
*tetA* + *tetB*	36 (34.61)	23 (22.12)	13 (12.50)
Total *tet* genes	68 (65.38)	37 (35.56)	31 (29.80)
	*Bla* genes		
*bla_ctx-_*_M_	22 (21.15)	1 (0.96)	20 (20.19)
*bla_TEM_*	9 (8.65)	3 (2.88)	6 (5.77)
*bla_SHV_*	10 (9.61)	3 (2.88)	7 (6.73)
*bla_ctx-_*_M_ + *bla_TEM_*	14 (13.46)	8 (7.69)	6 (5.77)
*bla_ctx-_*_M_ + *bla_SHV_*	3 (2.88)	1 (0.96)	2 (1.92)
*bla_SHV_* + *bla_TEM_*	3 (2.88)	1 (0.96)	2 (1.92)
*bla_ctx-_*_M_ + *bla_TEM_* + *bla_SHV_*	5 (4.80)	3 (2.88)	2 (1.92)
Total *bla* genes	58 (55.77)	20 (19.23)	45 (43.26)
	*Cat1* and *clmA* genes		
*cat1*	4 (3.85)	1 (0.96)	3 (2.88)
*clmA*	18 (17.31)	1 (0.96)	17 (16.35)
*cat1* + *clmA*	1 (0.96)	0	1 (0.96)
Total *cat1* and *clmA*	23 (22.12)	2 (1.92)	21 (20.19)

n = number of strains; % = percentage of the total of 104 strains.

**Table 4:** Genotypic antimicrobial resistance patterns of *Escherichia coli* isolates.

Isolates	Genes	n	%
1	*clmA–bla_CTX-M–_sul1–tetA–tetB–bla_TEM_–bla_SHV_*	1	0.96%
2	*bla_CTX-M–_sul1–tetA–tetB–bla_TEM_–bla_SHV_*	2	1.92%
3	*bla_CTX-M–_sul1–sul2–tetA–tetB–bla_SHV_*	1	0.96%
4	*bla_CTX-M–_sul1–sul2–tetA–tetB–bla_TEM_*	2	1.92%
5	*clmA–bla_CTX-M–_sul1–tetA–tetB–bla_SHV_*	1	0.96%
6	*bla_CTX-M–_sul1–sul2–tetA–bla_SHV_*	1	0.96%
7	*bla_CTX-M–_sul1–sul2–tetA–tetB*	1	0.96%
8	*bla_CTX-M–_sul1–tetA–bla_TEM–_bla_SHV_*	1	0.96%
9	*bla_CTX-M–_sul1–tetA–tetB–bla_SHV_*	1	0.96%
10	*bla_CTX-M–_sul1–tetA–tetB–bla_TEM_*	5	4.81%
11	*bla_CTX-M–_sul1–tetB–bla_TEM–_bla_SHV_*	1	0.96%
12	*cat1–bla_CTX-M–_sul1–tetB–bla_TEM_*	1	0.96%
13	*cat1–clmA–sul1–tetB–bla_TEM_*	1	0.96%
14	*clmA–bla_CTX-M–_sul1–tetA–tetB*	1	0.96%
15	*clmA–bla_CTX-M–_sul1–sul2–tetB*	1	0.96%
16	*clmA–bla_CTX-M–_tetA–tetB–bla_TEM_*	1	0.96%
17	*sul1–sul2–tetA–tetB–bla_SHV_*	1	0.96%
18	*sul1–sul2–tetA–bla_TEM_–bla_SHV_*	1	0.96%
19	*sul1–sul2–tetA–tetB–bla_SHV_*	1	0.96%
20	*sul1–tetA–tetB–bla_TEM_–bla_SHV_*	1	0.96%
21	*bla_CTX-M–_sul1–tetA–bla_TEM_*	1	0.96%
22	*bla_CTX-M–_sul2–tetA–tetB*	1	0.96%
23	*bla_CTX-M–_tetA–tetB–bla_TEM_*	1	0.96%
24	*bla_CTX-M–_sul1–tetB–bla_TEM_*	1	0.96%
25	*bla_CTX-M–_sul1–tetA–tetB*	2	1.92%
26	*cat1–bla_CTX-M–_sul1–tetA*	1	0.96%
27	*clmA–bla_CTX-M–_sul2–bla_TEM_*	1	0.96%
28	*clmA–bla_CTX-M–_tetA–tetB*	1	0.96%
29	*clmA–bla_CTX-M–_tetB–bla_TEM_*	1	0.96%
30	*clmA–sul1–tetA–bla_SHV_*	1	0.96%
31	*clmA–sul1–tetA–tetB*	1	0.96%
32	*clmA–tetA–tetB–bla_SHV_*	1	0.96%
33	*sul1–tetA–bla_TEM_–bla_SHV_*	1	0.96%
34	*sul1–tetA–tetB–bla_SHV_*	2	1.92%
35	*sul1–tetA–tetB–bla_TEM_*	3	2.88%
36	*bla_CTX-M–_sul1–sul2*	1	0.96%
37	*bla_CTX-M–_sul1–tetA*	3	2.88%
38	*bla_CTX-M–_tetA–bla_TEM_*	1	0.96%
39	*bla_CTX-M–_sul1–tetB*	1	0.96%
40	*bla_CTX-M–_tetA–tetB*	2	1.92%
41	*cat1–bla_CTX-M–_tetA*	1	0.96%
42	*cat1–sul1–bla_TEM_*	1	0.96%
43	*clmA–sul1–bla_TEM_*	1	0.96%
44	*clmA–sul1–tetA*	1	0.96%
45	*clmA–tetA–bla_TEM_*	1	0.96%
46	*sul1–tetA–tetB*	4	3.85%
47	*sul1–tetB–bla_SHV_*	1	0.96%
48	*sul1–tetB–bla_TEM_*	1	0.96%
49	*bla_CTX-M–_bla_SHV_*	1	0.96%
50	*bla_CTX-M–_sul2*	1	0.96%
51	*bla_CTX-M–_bla_TEM_*	1	0.96%
52	*bla_CTX-M_–tetB*	1	0.96%
53	*bla_CTX-M_–tetA*	1	0.96%
54	*clmA–bla_CTX-M_*	3	2.88%
55	*clmA–sul2*	1	0.96%
56	*sul1–bla_SHV_*	3	2.88%
57	*sul1–bla_TEM_*	2	1.92%
58	*sul1–tetA*	3	2.88%
59	*sul2–tetA*	1	0.96%
60	*tetA*	2	1.92%
Total		84	80.77%

**Table 5:** Primers sequences and amplified product sizes for the identification of *Escherichia coli*.

Gene	Primer Sequence (5′ → 3′)	Product Size (bp)	Reference
*lacZB*	F: ATGAAAGCTGGCTACAGGAAGGCC R: CACCATGCCGTGGGITICAATATT	876	Molina et al. [27]
*uidA*	F: TGGTAATTACCGACGAAAACGGC R: ACGCGTGGTTACAGTCTTGCG	162	Molina et al. [27]

F: foward (5′→3′); R: reverse (5′→3′); bp: base pairs.

**Table 6:** Antimicrobials, concentrations used, and reference values for the interpretation of inhibition zones in the antimicrobial susceptibility test for *Escherichia coli*.

Antimicrobials	Concentration	Class	S (mm)	I (mm)	R (mm)
Amoxicillin * (AMC)	20 + 10 μg	β-lactam/Penicillin	≤13	14–17	≥18
Cefotaxime (CTX)	30 μg	β-lactam/Cephalosporin	≤22	23–25	≥26
Ceftazidime (CAZ)	30 μg	β-lactam/Cephalosporin	≤17	18–20	≥21
Ciprofloxacin (CIP)	5 μg	Quinolone	≤21	22–25	≥26
Chloramphenicol (CLO)	30 μg	Phenicol	≤12	13–17	≥18
Gentamicin (GEN)	10 μg	Aminoglycoside	≤12	13–14	≥15
Imipenem (IMP)	10 μg	β-lactam/Carbapenem	≤19	20–22	≥23
Sulfonamide (SUL)	300 μg	Sulfonamide	≤13	14–17	≥18
Tetracycline (TET)	30 μg	Tetracycline	≤22	23–25	≥26

* Amoxicillin and clavulanic acid; μg: microgram; R: resistant; I: intermediate; S: susceptible; mm: millimeter.

**Table 7:** Primer sequences for the detection of antimicrobial resistance genes in *Escherichia coli* and PCR conditions.

Gene	Primer Sequence (5′ → 3′) F and R	bp	PCR Conditions	Reference
*sul1*	CTTCGATGAGAGCCGGCGGC GCAAGGCGGAAACCCGCGCC	238	Initial denaturation at 94 °C for 5 min; 30 cycles of denaturation at 94 °C for 60 s; annealing at 56 °C for 60 s; extension at 68 °C for 60; 72 °C for 10 min for final extension	Qiu et al. [59]
*sul2*	GCGCTCAAGGCAGATGGCATT GCGTTTGATACCGGCACCCGT	293	Initial denaturation at 95 °C for 10 min; 35 cycles of denaturation at 94 °C for 45 s; annealing at 55 °C for 50 s; extension at 72 °C for 50; 72 °C for 10 min for final extension	Arabi et al. [60]
*tetA*	GGCGGTCTTCTTCATCATGC CGGCAGGCAGAGCAAGTAGA	502	Initial denaturation at 94 °C for 60 s; 30 cycles of denaturation at 95 °C for 60 s; annealing at 55 °C for 60 s; extension at 72 °C for 60 s; 72 °C for 8 min for final extension	Belaynehe et al. [23]
*tetB*	TTGGTTAGGGGCAAGTTTTG GTAATGGGCCAATAACACCG	659	Initial denaturation at 94 °C for 60 s; 30 cycles of denaturation at 95 °C for 30 s; annealing at 55 °C for 30 s; extension at 72 °C for 60 s; 72 °C for 8 min for final extension	Ahmed et al. [61]
*bla_ctx-_*_M_	ATGTGCAGYACCAGTAARGTKATGGC TGGGTRAARTARGTSACCAGAAYCAGCGG	592	Initial denaturation at 94 °C for 60 s; 36 cycles of denaturation at 94° C for 30 s; annealing at 58 °C for 60 s; extension at 72 °C for 60 s; 72 °C for 10 min for final extension	Boyd et al. [62]
*bla_TEM_*	TTGGGTGCACGAGTGGGTTA TAATTGTTGCCGGGAAGCTA	506	Initial denaturation at 95 °C for 3 min; 35 cycles of denaturation at 94 °C for 60 s; annealing at 55 °C for 60 s; extension at 72 °C for 1 s; 72 °C for 7 min for final extension	Gundran et al. [58]
*bla_SHV_*	TCGGGCCGCGTAGGCATGAT AGCAGGGCGACAATCCCGCG	628	Initial denaturation at 95 °C for 3 min; 35 cycles of denaturation at 94 °C for 60 s; annealing at 55 °C for 60 s; extension at 72 °C for 1 s; 72 °C for 7 min for final extension	Gundran et al. [63]
*cat1*	AGTTGCTCAATGTACCTATAACC TTGTAATTCATTAAGCATTCTGCC	547	Initial denaturation at 94 °C for 5 min; 30 cycles of denaturation at 94 °C for 30 s; annealing at 50 °C for 30 s; extension at 72 °C for 1 s; 72 °C for 10 min for final extension	Van et al. [64]
*clmA*	CCGCCACGGTGTTGTTGTTATC CACCTTGCCTGCCCATCATTAG	698	Initial denaturation at 94 °C for 5 min; 30 cycles of denaturation at 94 °C for 30 s; annealing at 50 °C for 30 s; extension at 72 °C for 1 s; 72 °C for 10 min for final extension	Van et al. [64]

F: foward (5′→3′); R: reverse (5′→3′); bp: base pairs.

## Data Availability

The data presented in this study are available on request from the corresponding author. The data is not publicly available due to privacy interests.
